# Utility of F18-FDG PET/CT in the Evaluation of Pituitary Uptake

**DOI:** 10.1055/s-0044-1787967

**Published:** 2024-06-25

**Authors:** Adersh Stanly, Saumya Sara Sunny, Justin Benjamin, Hesarghatta Shyamasunde Asha, David Mathew, Junita Rachel John, Julie Hephzibah

**Affiliations:** 1 Department of Nuclear Medicine, Christian Medical College and Hospital, Vellore, Tamil Nadu, India; 2 Department of Endocrinology, Christian Medical College and Hospital, Vellore, Tamil Nadu, India

**Keywords:** F18-FDG PET/CT, hypophysitis, macroadenoma, pituitary adenoma, pituitary uptake

## Abstract

**Introduction** Pituitary adenoma is the most common disease that affects the gland and may be classified as functional/nonsecretory tumors. Inflammatory/infective causes may also affect the pituitary gland. The 18F-fluorodeoxyglucose positron emission tomography/computed tomography (F18-FDG PET/CT) may have an incremental value in assessing these lesions and in determining their clinical significance.

**Aim** This article assesses the utility of F18-FDG PET/CT in detecting and determining clinical profile of pituitary lesions with abnormal uptake.

**Methodology** Retrospective analysis of all patients who underwent F18-FDG PET/CT from January 2015 to January 2023 was done. Those with abnormal pituitary uptake (standardized uptake value [SUV] > 2.5) were included in the study. SUV value along with relevant anatomical details, biochemical parameters, histopathological details, and follow-up imaging were analyzed.

**Results** Among 15,085 studies, a total of 36 patients (21 males/15 females, average age 47.36 years, range: 17–75 years) with pituitary uptake (0.23%) were included. Out of 36 patients, causes are primary pituitary tumor (21/36, 58%), tubercular hypophysitis (3/36, 8%), lymphocytic hypophysitis (2/36, 6%), lymphomatous involvement (2/36, 6%), autoimmune hypophysitis (1/36, 3%), questionable significance/incidental (4/36, 11%), and metastasis (3/36, 8%)—one each from neuroendocrine tumor ileum, chondrosarcoma, and adenocarcinoma lung. There was no difference in the SUV range between the different etiologies.

Among 21 patients with pituitary tumor, biochemical evaluation was done in 19 patients. Two patients were lost to follow-up and did not have biochemical evaluation. Among them, 8 underwent endoscopic transsphenoidal radical excision and 1 patient had PET-CT-guided stereotactic radiosurgery alone. In another 8 patients who had prior endoscopic transsphenoidal radical excision, uptake was noted as residual lesion on PET-CT. Of them, 3 underwent subtotal excision and 5 had PET-CT-guided stereotactic radiosurgery. Biopsy was done in 14 patients, of which 11 were macroadenoma and 3 were microadenoma. Overall, magnetic resonance imaging (MRI) brain was performed in 22 of them and the findings were concordant with F18-FDG PET/CT.

**Conclusion** F18-FDG PET/CT is a useful modality in the evaluation of pituitary uptake. It has an incremental value along with MRI brain and biochemical parameters and is useful for follow-up. Due to its high diagnostic accuracy, it is particularly useful in those with suspected residual/recurrent adenomas.

## Introduction

### Pituitary Gland and Causes Leading to Its Abnormality

The pituitary gland is positioned near the base of the brain and is connected to the inferior hypothalamus and weighs less than half a gram. The pituitary gland is known as the “master gland” because it governs essential hormone output. 1 Function of the pituitary gland can be compromised due to neoplastic, inflammatory lesions or rarely infections. Among these, pituitary adenomas are the most common, slow-growing, benign tumors of the anterior pituitary, which can be classified as microadenoma, macroadenoma, or giant tumors. 2 The Central Brain Tumor Registry reports that pituitary adenomas make up 16.9% of all brain tumors, despite of inconsistent prevalence estimates. 3

Pituitary microadenoma is an incidental finding and patients will be usually asymptomatic, while pituitary macroadenoma presents with mass effects and potential hormonal deficiency or excess. 2 Of pituitary adenomas, around 40% are nonfunctioning. 4 Prolactin-secreting adenoma is a condition where elevated prolactin levels suppress gonadotropin secretion, leading to infertility, decreased libido, and osteoporosis in both sexes. It can cause amenorrhea, galactorrhea, erectile dysfunction, and gynecomastia in women. Growth hormone (GH)-secreting adenoma (acromegaly) presents with headaches, vision changes, arthritis, carpal tunnel syndrome, and excessive sweating. Patients may also have coarse facial features, frontal bossing, enlarged nose, prognathism, enlarged tongue, and skin tags. Adrenocorticotropic hormone (ACTH)-secreting adenoma (Cushing's disease) presents with weight gain, muscle weakness, mood disorders, easy bruising, and multiple fractures. In thyroid-stimulating hormone (TSH) secreting adenoma patients may experience symptoms of thyrotoxicosis like palpitations, arrhythmias, weight loss, tremors, and a goiter. 2 Although pituitary gland metastases are uncommon, they should be suspected in cases of rapidly progressing tumors, hyperprolactinemia, headache, cranial nerve palsies, or rapid onset diabetes insipidus. 5

Primary pituitary infections occur in normal pituitary glands, while secondary infections occur in patients with preexisting pituitary lesions like pituitary adenoma, Rathke's cleft cyst, craniopharyngioma, or following surgery. Bacterial infections, like mycobacterium tuberculosis, can cause pituitary inflammation leading to secondary hypophysitis. 6

### Treatment Options of Pituitary Adenoma

Dopamine agonists (DAs) like cabergoline and bromocriptine are the first-line treatment for prolactin-secreting tumors, with cabergoline being over 90% effective in normalizing prolactin levels and decreasing tumor size. 2

Surgery is the preferred treatment for pituitary adenomas. 1 Endoscopic transsphenoidal surgery is the most common procedure for pituitary adenoma due to its safety and effectiveness. 7 Invasive pituitary adenomas are common, but complete surgical removal is not always possible, especially for macroadenomas with cavernous sinus extension. 8 Stereotactic radiotherapy is a viable treatment option for patients with persistent or recurrent pituitary adenomas after unsuccessful surgery, particularly if the tumor is enlarging or resistance to medical therapy is present. 9

### Role of Imaging

Pituitary imaging plays a crucial role when it comes to the differential diagnosis of various sellar lesions and the confirmation of the diagnosis of pituitary lesions. 10 Plain skull radiographs are infrequently used for diagnosing sellar and parasellar pathologies due to their inability to accurately delineate soft tissues and the lack of sensitivity to pituitary gland abnormality, leading to their replacement by cross-sectional imaging techniques like computed tomography (CT) scanning and magnetic resonance imaging (MRI). MRI is the preferred method for assessing hypothalamic-pituitary-related endocrine diseases, aiding in diagnosis, differentiation, and understanding the gland's relationship with adjacent structures, aiding in medical or surgical strategy planning. 11

### MRI—Is It a Gold Standard?

In recurrent pituitary tumors, structural findings are crucial for calculating radiation fields and monitoring drug therapy efficacy, especially when considering repeated operations. CT scan is limited in its ability to differentiate postoperative changes and recurrent pituitary tumors. 12 Currently, MRI is the gold standard for detecting pituitary adenomas. 13 However, the diagnostic accuracy is limited in locating small tumors or identifying tumor recurrence due to altered anatomy, postsurgery. 14 15 16 Postoperative MR images of the sella turcica region are challenging to interpret due to various factors such as the size and extent of the adenoma, the surgical approach, the quality and quantity of implanted materials, and the time course of resolution of postoperative changes. 17

### Pituitary and 18F-Fluorodeoxyglucose Positron Emission Tomography/Computed Tomography

Under physiological conditions, pituitary gland volume is typically small and appears as background level uptake in 18F-fluorodeoxyglucose (18F-FDG) positron emission tomography/CT (PET/CT) imaging. 18 The pituitary gland's small size and low metabolic rate may cause the accumulation of 18F-FDG in both benign and neoplastic pituitary lesions. 19 Pituitary uptake is typically linked to benign tumors, including nonfunctioning macroadenomas, but functional tumors can also be detected. Metastases should be considered in differential diagnosis. Pituitary metastases are rare, accounting for only 0.4% of intracranial metastases and approximately 1% of surgically treated pituitary lesions. 20 Primary tumors responsible for metastases are lung (36.8%), followed by breast (22.9%) and kidney (7.0%), with other malignancies including colon cancer, melanoma, non-Hodgkin lymphoma, and plasmacytoma. 20 21 More recently, a 77-year-old woman's endometrial cancer metastasis to the sellar/suprasellar area has been described. This metastasis was identified by 18F-FDG PET and confirmed thereafter by CT and MRI. 22 18F-FDG PET can detect pituitary metastasis, with a low prevalence of approximately 6%. 19

Inflammatory pituitary diseases like lymphocytic hypophysitis can also be detected using F18-FDG PET/CT. 19 18F-FDG PET can detect primary lymphocytic hypophysitis and secondary hypophysitis, including immunotherapy-induced hypophysitis in cancer patients. 23 24 25 In a recent study, 18F-FDG PET/CT revealed ipilimumab-induced hypophysitis in a 77-year-old man with advanced melanoma, and after 4 weeks of prednisolone therapy, the uptake of 18F-FDG in the pituitary gland normalized. 23

Pituitary adenoma, characterized by metabolically active cells, shows significantly increased 18F-FDG uptake. 12 The 18F-FDG uptake rate in pituitary adenoma is 30% higher than in the whole brain, and it is higher in nonfunctional pituitary adenomas compared with functioning ones. Recurrent macroadenomas have similar metabolism to untreated adenomas, and glucose uptake is lower in irradiated tumors. Medical therapy with DAs or somatostatin analogs decreases glucose utilization. 18F-FDG PET/CT has been found to have higher sensitivity in detecting pituitary adenoma compared with contrast-enhanced MRI, particularly in patients with small lesions or potential recurrence. 26

This study is done to look for the incremental value of F18-FDG PET/CT in assessing pituitary lesions and determining their clinical significance, especially in cases of recurrent pituitary adenomas.

### Methodology

Retrospective analysis of patients who visited the nuclear medicine department from January 2015 to January 2023 was done. All of them had F18-FDG PET/CT for various indications.

Of these, patients with pituitary uptake > 2.5 standardized uptake value (SUV) were included.

SUV value along with relevant anatomical details, biochemical parameters, histopathological details, treatment details, and follow-up imaging were analyzed. Hormone assays such as plasma ACTH, serum cortisol, prolactin, GH, follicle-stimulating hormone (FSH), luteinizing hormone (LH), and insulin-like growth factor 1 (IGF-1) values which were done as a part of pituitary adenoma workup were analyzed. MRI brain of these patients was analyzed along with the treatment details. Biopsy report of the patients who underwent surgery for pituitary adenoma was also examined.

## Results

A total of 15,085 patients underwent F18-FDG PET/CT during the study period from January 2015 to January 2023. Of these, 36 patients (0.23%) fulfilled the inclusion criteria of SUV > 2.5. There were 21 males and 15 females. The average age was 47.36 years (range: 17–75 years). The median age among male was 52 years and among female 40.8 years. The mean SUVmax was 10.23 ± 7.36 (range: 3.35–30.8). There was no difference in the SUV range between different etiologies.

A total of 22 patients underwent MRI brain imaging prior to or after the F18-FDG PET/CT. The findings of the MRI brain were consistent with PET-CT findings.

**Fig. 1:**
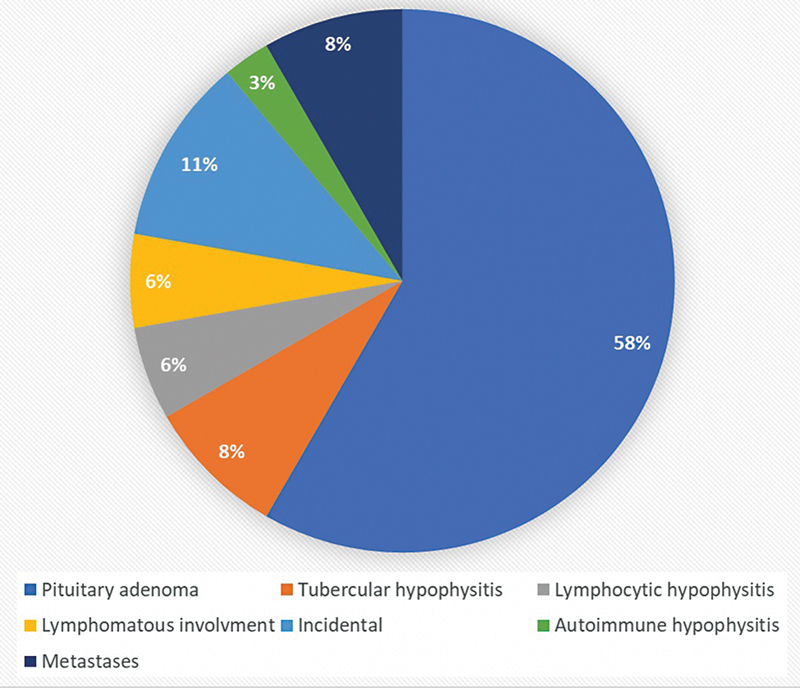
Cause of pituitary uptake.

The causes of pituitary uptake in the descending order of prevalence were primary pituitary tumor (21/36, 58%), tubercular hypophysitis (3/36, 8%), lymphocytic hypophysitis (2/36, 6%), lymphomatous involvement (2/36, 6%), autoimmune hypophysitis (1/36, 3%), questionable significance/incidental (4/36, 11%), and metastasis (3/36, 8%)—one each from neuroendocrine tumor ileum, chondrosarcoma, and adenocarcinoma lung ( Fig. 1 ).

The 21 patients with primary pituitary adenoma had a SUVmax ranging from 3.35 to 30.8 (10.31 ± 7.85).

Biochemical evaluation was done in 19 patients. Two patients were lost to follow-up and did not have biochemical evaluation. According to biochemical evaluation, 6 patients had GH-secreting pituitary adenoma, 4 patients had ACTH-secreting pituitary adenoma, 3 patients had GH-secreting pituitary adenoma and secondary hypothyroidism, 1 patient had FSH-secreting pituitary adenoma, 1 with hypogonadotropic hypogonadism and secondary hypocortisolism, 1 with ACTH-secreting pituitary adenoma and secondary hypothyroidism, 1 patient had GH-secreting adenoma with secondary hypothyroidism, hypocortisolism, and hypogonadism, and 2 patients had normal pituitary function ( Table 1 ).

**Table 1:** Pituitary adenoma patients—SUVmax and biochemical evaluation

Age	Gender	Biochemical evaluation	SUVmax
31	M	No evaluation	10.2
62	M	Hypogonadotropic hypogonadism, secondary hypocortisolism	30.8
61	M	Normal pituitary function	22.06
49	M	ACTH-secreting pituitary adenoma	3.65
48	M	Growth hormone-secreting pituitary tumor	3.63
56	M	Growth hormone-secreting pituitary tumor	13.1
75	M	No evaluation	12.01
45	M	Growth hormone-secreting pituitary tumor and secondary hypothyroidism	6.78
51	M	Growth hormone-secreting pituitary tumor	6.36
21	M	Growth hormone-secreting pituitary tumor	4.08
56	M	FSH-secreting pituitary adenoma	5.05
27	M	Growth hormone-secreting pituitary adenoma and secondary hypothyroidism	7.69
32	M	Growth hormone-secreting pituitary tumor	3.35
53	F	Normal pituitary function	19.49
32	F	ACTH-secreting pituitary adenoma	7.12
42	F	Growth hormone-secreting pituitary tumor and secondary hypothyroidism	5.19
26	F	ACTH-secreting pituitary adenoma	4.19
17	F	ACTH-secreting pituitary adenoma	7.9
28	F	ACTH-secreting pituitary adenoma and secondary hypothyroidism	8.04
31	F	Growth hormone-secreting pituitary tumor and secondary hypothyroidism, hypocortisolism, and hypogonadism	3.68
39	F	Growth hormone-secreting pituitary tumor	5.04

Abbreviations: ACTH, adrenocorticotropic hormone; F, female; FSH, follicle-stimulating hormone; M, male; SUVmax, maximum standardized uptake value.

**Fig. 2:**
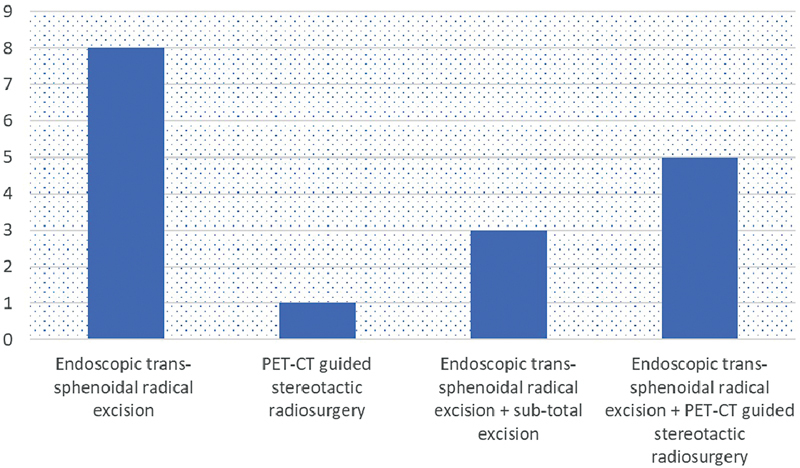
Treatment of pituitary tumor-positive positron emission tomography (PET) uptake.

A total of 17/21 patients who were diagnosed with pituitary tumor underwent different forms of treatment ( Fig. 2 ). Of which 16 of them underwent endoscopic transsphenoidal radical excision and 1 underwent PET-CT-guided stereotactic radiosurgery alone. All individuals have undergone surgery and have been confirmed through pathologic examination with immunohistochemistry. Three of them were gonadotroph adenoma, 3 corticotropic adenoma, 3 GH and prolactin producing pituitary adenoma, 2 were plurihormonal pituitary adenoma, 4 with atypical sparsely granulated GH-producing pituitary adenoma, and 1 patient was not categorized into it.

Among 8 patients who had prior endoscopic transsphenoidal radical excision, F18-FDG uptake was noted which was suggestive of residual lesion/recurrence. Among these patients, 3 underwent subtotal excision and 5 had PET-CT-guided stereotactic radiosurgery.

## Discussion

The pituitary gland is often seen as background activity in 18F-FDG PET/CT imaging due to its small size and low metabolic rate. 18 27 In the current study, it is demonstrated that 0.23% of the total patients showed pituitary uptake. The incidence of pituitary uptake is almost comparable with the study by Ju et al who reported that incidence is 0.13%. 28 Incidence of pituitary uptake in our study is consistent with the study by Hyun et al, who reported that incidence of pituitary uptake is 0.8% and the study by Jeong et al, who reported the incidence to be 0.073%. 21 29 The difference may be due to a different patient population.

**Fig. 3:**
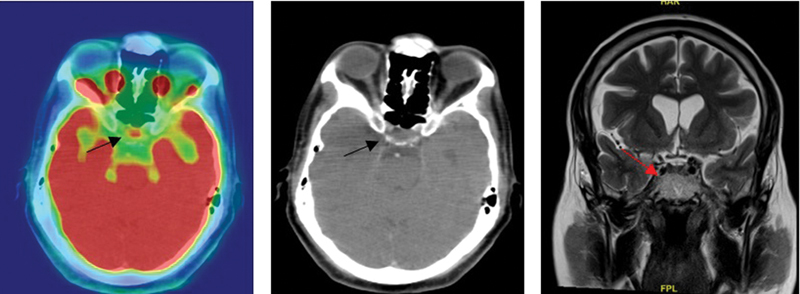
A 52-year-old male with disseminated tuberculosis—pituitary involvement (standardized uptake value [SUV] 5.5). Magnetic resonance imaging (MRI) brain showed enlarged pituitary gland with enhancing sellar surpraseller lesion involving the pituitary stalk. Follow-up MRI showed resolution of the lesion.

The most common cause of pituitary uptake from our study is primary pituitary tumor (58%) followed by tubercular hypophysitis (8%) ( Fig. 3 ), lymphocytic hypophysitis (6%), lymphomatous involvement (6%), autoimmune hypophysitis (%), questionable significance/incidental (4/36, 11%), and metastasis (3/36, 8%)—one each from neuroendocrine tumor ileum, chondrosarcoma, and adenocarcinoma lung. Seok et al's study revealed that 79% of patients with pituitary uptake have pituitary adenomas similar to our study. 30

SUVmax of patients with primary pituitary adenoma ranged from 3.35 to 30.8 (10.31 ± 7.85). The mean SUVmax is comparable to previous studies by Jeong et al and Hyun et al, which reported a mean of 11.5 ± 8.4 and 10.9 ± 7.0, respectively. 21 29

In comparison with the study by Ju et al, where 3 out of 9 patients had no abnormal laboratory findings, 28 our study had 19 patients with abnormal biochemical values. Six patients had GH-secreting pituitary adenoma, 4 patients had ACTH-secreting pituitary adenoma, 3 patients had GH-secreting pituitary adenoma and secondary hypothyroidism, 1 patient had FSH-secreting pituitary adenoma, 1 with hypogonadotropic hypogonadism and secondary hypocortisolism, 1 with ACTH-secreting pituitary adenoma and secondary hypothyroidism, 1 patient had GH-secreting adenoma with secondary hypothyroidism, hypocortisolism, and hypogonadism, and 2 patients had normal pituitary function. Thus, F18-FDG PET/CT, along with other biochemical parameters, plays an essential role in diagnosing pituitary adenomas.

Among 23 patients diagnosed with pituitary tumors, 17 patients underwent different treatment methods, including endoscopic transsphenoidal radical excision and PET-CT-guided stereotactic radiosurgery. All patients underwent surgery and were confirmed through immunohistochemistry. The patients were classified into gonadotroph, corticotropic, GH- and prolactin-producing, plurihormonal, and atypical sparsely granulated GH-producing adenoma.

**Fig. 4:**
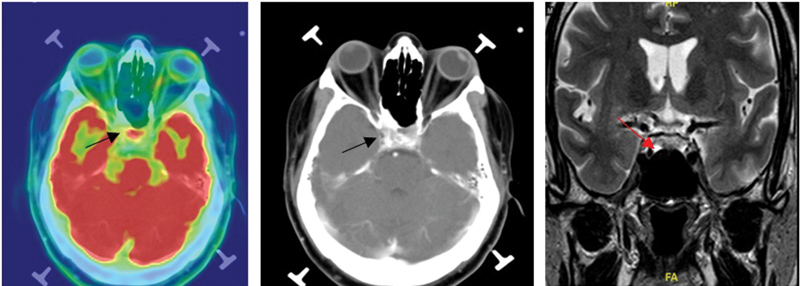
A 51-year-old male with pituitary adenoma recurrence. Initially underwent endoscopic transsphenoidal radical excision. Histopathology showed corticotroph adenoma, MIB-1 labeling index ∼6–8%, and biochemically growth hormone-secreting pituitary adenoma. Follow-up magnetic resonance imaging (MRI) showed mild distortion of the sellar region. Underwent 18F-fluorodeoxyglucose positron emission tomography/computed tomography (F18-FDG PET/CT), showed pituitary uptake with an maximum standardized uptake value (SUVmax) of 3.35. As recurrence, underwent endoscopic transsphenoidal subtotal excision and biopsy confirmed atypical sparsely granulated growth hormone-producing pituitary adenoma.

F18-FDG PET/CT is highly effective for diagnosing recurrence or residual pituitary lesions, while MRI brain may be limited in localizing small tumors or identifying tumor recurrence due to altered anatomy postsurgery. 14 15 16 In our study, despite a small number of recurrent or residual lesions, all lesions exhibited pituitary uptake. An initial MRI of a 51-year-old gentleman showed T2 hypointense enhancing solid mass in widened sella in the central part and left lateral aspect with mild suprasellar and posterolateral extension and underwent endoscopic transsphenoidal excision. Histopathology showed corticotroph adenoma with MIB-1 labeling index of approximately 6 to 8%. As symptoms were persisting, a follow-up MRI was taken and showed mild distortion of the sellar region. Biochemical evaluation showed GH-secreting adenoma. The patient underwent F18-FDG PET/CT, showed pituitary uptake with an SUVmax of 3.35, and underwent endoscopic transsphenoidal subtotal excision and biopsy confirmed atypical sparsely granulated GH-producing pituitary adenoma ( Fig. 4 ).

Pituitary inflammation typically has a positive prognosis after standard hormone treatment, making invasive operations unnecessary. Currently, reliable diagnosis requires integrating various examinations without unnecessary pituitary biopsy. Patients have to undergo initial pituitary function testing, including cortisol, TSH, prolactin, LH, FSH, FSH with estradiol or testosterone, IGF-1, and serum sodium, plasma, and urine osmolarity, to assess for any abnormality in pituitary. 31 Depending on the clinical context and etiology, patient will undergo other blood investigations. The baseline assessment includes blood count, C-reactive protein, calcium, creatinine, urinalysis, and alanine transaminase, with specific investigations tailored to clinical features like immunoglobulin G4, angiotensin-converting enzyme, antineutrophil cytoplasmic antibodies, antinuclear antibody, lactate dehydrogenase, β2-microglobulin, α-fetoprotein, human chorionic gonadotropin, tuberculin skin test, or QuantiFERON-TB Gold. 32 PET/CT-based diagnoses of lymphatic pituitary inflammation are rarely adopted. 28 A case report showed pituitary hypermetabolism in a melanoma patient with ipilimumab treatment, which was later diagnosed as lymphatic pituitary inflammation. 23 In our study, three patients had tubercular hypophysitis and were treated with conservative management and antitubercular treatment. Following this, patients responded well. A patient with skull base osteomyelitis and associated hypophysitis demonstrated good outcomes after receiving proper medical management.

Various PET tracers can be used to detect pituitary uptake. 68Ga-DOTA-TATE PET/CT has been used to identify pituitary micro- and macroadenomas that are either nonfunctional or functioning. But in contrast to 18F-FDG, 68Ga-DOTA-TATE often appears to be more helpful in identifying any residual normal pituitary tissue, whereby 18F-FDG exhibits a higher capacity to identify recurring or residual pituitary adenomas following transsphenoidal excision. 33 Pituitary uptake on 18F-DOPA PET has been positive in some pituitary adenomas, mainly prolactinomas. Medical treatment with DA is the preferred treatment for prolactinoma, achieving normalization in 73 to 96% of patients and decreasing tumor size in 50 and 100%. 34 Other tracers include 18F-choline, 11C-methionine, 18F-FET (O- (2–18F-fluoroethyl)-l-tyrosine), and 13N-ammonia. 35 36 37 Another potential molecular imaging method for identifying ACTH-dependent microadenoma is 68Ga-DOTA CRH (corticotrophin-releasing hormone) PET/CT. 38 68Ga CRH PET imaging offers a comprehensive view of the entire body, including adrenal glands, detecting ectopic ACTH-secreting lesions, and adrenal gland abnormalities like adenomas. 68Ga CRH PET-CT targets CRH receptors, identifying corticotropinoma and providing surgeons with valuable information for intraoperative tumor navigation and distinguishing pituitary and extrapituitary sources of ACTH-dependent Cushing's syndrome. 39

The limitations of our study include its small study sample size from a single institution and its retrospective design. All foci of uptake were not confirmed with biopsy.

## Conclusion

F18-FDG PET/CT is a useful modality in the evaluation of pituitary uptake. It has an incremental value along with MRI brain and biochemical parameters and is useful for follow-up. Due to its high diagnostic accuracy, it is particularly useful in those with suspected residual/recurrent adenomas.
